# The Beneficial Effects of Edible Kynurenic Acid from Marine Horseshoe Crab (*Tachypleus tridentatus*) on Obesity, Hyperlipidemia, and Gut Microbiota in High-Fat Diet-Fed Mice

**DOI:** 10.1155/2021/8874503

**Published:** 2021-05-03

**Authors:** Jian Li, Yaqi Zhang, Shen Yang, Zhenhua Lu, Guiling Li, Shangyi Wu, Da-Ren Wu, Jingwen Liu, Bo Zhou, Hui-Min David Wang, Shi-Ying Huang

**Affiliations:** ^1^College of Food and Biological Engineering, Jimei University, Xiamen 361021, China; ^2^Fujian Provincial Engineering Technology Research Center of Marine Functional Food, Xiamen 361021, China; ^3^Xiamen Bioendo Technology Co., Ltd., Xiamen 361022, China; ^4^Department of Microbiology, College of Life Sciences, Shandong Agricultural University, Tai'an 271018, China; ^5^Graduate Institute of Biomedical Engineering, National Chung Hsing University, Taichung 402, Taiwan; ^6^Graduate Institute of Medicine, College of Medicine, Kaohsiung Medical University, Kaohsiung 807, Taiwan; ^7^Department of Medical Laboratory Science and Biotechnology, China Medical University, Taichung 404, Taiwan

## Abstract

The marine horseshoe crab (*Tachypleus tridentatus*) has been considered as food and traditional medicine for many years. Kynurenic acid (KA) was isolated from horseshoe crab in this study for the first time in the world. A previous study in 2018 reported that intraperitoneal administration of KA prevented high-fat diet- (HFD-) induced body weight gain. Now, we investigated the effects of intragastric gavage of KA on HFD mice and found that KA (5 mg/kg/day) inhibited both the body weight gain and the increase of average daily energy intake. KA reduced serum triglyceride and increased serum high-density lipoprotein cholesterol. KA inhibited HFD-induced the increases of serum low-density lipoprotein cholesterol, coronary artery risk index, and atherosclerosis index. KA also suppressed HFD-induced the increase of the ratio of Firmicutes to Bacteroidetes (two dominant gut microbial phyla). KA partially reversed HFD-induced the changes in the composition of gut microbial genera. These overall effects of KA on HFD mice were similar to that of simvastatin (positive control). But the effects of 1.25 mg/kg/day KA on HFD-caused hyperlipidemia were similar to the effects of 5 mg/kg/day simvastatin. The pattern of relative abundance in 40 key genera of gut microbiota from KA group was closer to that from the normal group than that from the simvastatin group. In addition, our *in vitro* results showed the potential antioxidant activity of KA, which suggests that the improvement effects of KA on HFD mice may be partially associated with antioxidant activity of KA. Our findings demonstrate the potential role of KA as a functional food ingredient for the treatment of obesity and hyperlipidemia as well as the modulation of gut microbiota.

## 1. Introduction

Horseshoe crab (*Tachypleus tridentatus*) has been known as “living fossils” [1], which could date back over 445 million years [2, 3]. Horseshoe crab, one of the most important marine organisms for edible-medicinal use, has high scientific research value [4, 5]. The blood products of horseshoe crabs have been widely used to detect endotoxin [6]. Moreover, horseshoe crabs have been often used in traditional medicine and regional food culture for a long time period in the past in the Asian region, including the tail of horseshoe crab. At present, the research on horseshoe crab mainly focuses on *Tachypleus* Amebocyte Lysate (TAL), tachyplesin, and the technology of extracting chitin from horseshoe crab shells. However, there are few reports on the composition and chemical properties of horseshoe crab. To the best of our knowledge, kynurenic acid (KA) was isolated from horseshoe crab tail for the first time in this study.

The canine urine was identified to have KA in 1853 [7]. KA is the metabolite of tryptophan through the kynurenine pathway, and KA is the nonselective antagonist for ionotropic glutamate receptors [8, 9]. Several types of tissues produce KA endogenously, including brain tissue [10] and retina [11]. The physiological fluids could be detected to have KA, such as urine, serum [12], and cerebrospinal fluid [10]. The role of KA in the brain at physiological and pathological states has been excessively studied. But in the periphery, the role of KA is not entirely elucidated yet. Previous researches have demonstrated that antioxidative [13], anti-inflammatory [14, 15], antiatherogenic [16], analgesic [17], antiulcerative [18], and hepatoprotective [19] effects of KA. Recent reports further suggested that KA regulates adipose tissue energy homeostasis through G protein-coupled receptor Gpr35-mediated pathway [20], and KA is a endogenous aryl hydrocarbon receptor ligand [21]. However, the role of KA as a functional food ingredient for obesity and hyperlipidemia still remains unclear.

Hyperlipidemia and its complications affect many people, and the direct manifestation is obesity. It has been one of the primary reasons of untimely death and one of the key reasons of mortality for both developed and developing nations [22]. Hyperlipidemia has been considered as a metabolic disorder of systemic lipids, especially total cholesterol (TC), triglyceride (TG), low-density lipoprotein cholesterol (LDL-C), and high-density lipoprotein cholesterol (HDL-C). During hyperlipidemia, plasma lipids could be increased (in TC, TG, or LDL-C) or decreased (in HDL-C) compared with normal condition. It has been also regarded as one of the major hazard factors for causing cardio-cerebrovascular diseases, including atherosclerosis and stroke. For patients with cardiovascular diseases, the mortality of patients with hyperlipidemia was highly increased [23]. Epidemiological scientists have reported that long-term intake of antioxidative foods has the potential for protection against metabolic and cardiovascular diseases [24]. Thus, development of new functional foods has been urgently needed for improving hyperlipidemia.

Compared with other areas in human, gut microbiota has both the largest numbers of bacteria and the highest number of species types. Gut microbiota is mainly composed of intestinal flora, and there are about 10^14^ colony-forming unit (CFU) in the human intestine. Gut microbiota has a key regulatory role for the body's metabolism and is highly related with human diseases, including atherosclerosis [25–27]. Gut microbiota affects the ingestion behaviors according to the preferences of microbiota leading the obesity, due to consume extra food ultimately from the host [28]. With more microbiome diversity in gut, the microbiota spend greater resources and energy in competing with other microbiota and have lower influence on the host [29]. The opposite evidence is that with less gut microbiome diversity, and microbiota labor together to create energy desires in the host. But, to our knowledge, no earlier studies about the possible effects of KA on gut microbiota have been reported.

Based on the two previous studies about KA, we speculated that oral KA might have the beneficial effects on high-fat diet (HFD) model. First, a previous study in mice in 2018 reported that the intraperitoneal (i.p.) administration of KA (5 mg/kg/day) prevents body weight gain induced by HFD [20]. But the possible effects of oral KA on HFD model still need to be explored in this study. Second, a previous study has demonstrated the antioxidative effect of KA [13]. Feed of a universal antioxidant lipoic acid reduced HFD-induced oxidative stress in mice with improvement of gut microbiota [30], which implied that KA might have the similar effects. Therefore, we designed a series of experiments for testing our hypothesis.

In this study, the compound of KA was isolated and purified from horseshoe crab tail by modern spectral analysis methods, such as TLC, UV, NMR, and ESI-MS. The antioxidant activities of KA from horseshoe crab were determined. The effects of KA on food intake, energy intake, body weight, and serum lipids were investigated in the HFD mouse model. This study also explored the possible effects of KA on gut microbiota in HFD mice. We selected simvastatin (SV) as a positive control [31].

## 2. Materials and Methods

### 2.1. Materials and Chemicals

Horseshoe crab (*Tachypleus tridentatus*) was collected from the North Gulf, Xiamen, China. Reversed phase silica gel YMC-ODS C18 was purchased from YMC (Kyoto, Japan). Silica gel chromatography plates GF254 were obtained from Qingdao Ocean Chemical Plant (Qingdao, China). We prepared normal phase column chromatography silica gel (Silia Flash P60) from SiliCycle (Quebec City, Canada), Sephadex LH-20 from GE Healthcare (Uppsala, Sweden), and deuterated solvents for NMR experiments from Shanghai Macklin Biochemical Co. Ltd. (Shanghai, China). Methanoic acid and methanol for ESI-MS were purchased from TEDIA Biochemical Co. Ltd. (USA). For FRAP and ABTS assay, we used total antioxidant capacity assay kit from Beyotime Institute of Biotechnology (Shanghai, China). We acquired all other reagents with analytical grade from Sinopharm Chemical Reagent Co. (Shanghai, China).

### 2.2. Preparation of the Crude Extract

The dried horseshoe crab tail was crushed and further extracted by three times with methanol in a ratio of 1 : 10 (*w*/*v*) at 40°C for 24 h. The extracts were combined, concentrated, and evaporated for dryness by a rotary evaporator (N-1100, EYELA, Japan) at 45°C. The methanol extract of horseshoe crab tail was dissolved in a little amout of methanol and then filtered using a Dismic-25 JP membrane filter with pore diameter of 0.45 *μ*m (Advantec Toyo, Tokyo, Japan). Finally, the filtrate was evaporated to concentrate the crude extract.

### 2.3. Separation and Purification of the Compound

The methanol extract was separated into 4 fractions by silica gel column chromatography (3.6 × 25 cm) with gradient elution of water and methanol from (1 : 0 to 0 : 1). The fraction 2 was applied to Sephadex LH-20 column chromatography (2.5 × 150 cm) with methanol. The fraction was subjected to TLC analysis and then to reverse silica gel column chromatography by gradient elution of water and acetone from 4 : 6 to 0 : 1, which was separated into fraction A and fraction B. The fraction B was repeatedly recrystallized three times to finally obtain the compound.

### 2.4. Structural Identification of the Compound

The purified component HCT8 was dissolved in a trace amount of methanol and placed in a clean nuclear magnetic tube. The methanol was subjected to rotary evaporation with a rotary evaporator; the dry weight was weighed and labeled and then dissolved with the corresponding deuterated reagent. We recorded ^1^H-NMR, ^13^C-NMR, ^1^H-^1^H COSY, HMBC, and HSQC (400 MHz) spectra of the compound by a Bruker AVANCE-400 NMR spectrometer (Bruker Co., Rheinstetten, Germany). The mass spectra was analyzed by ESI-MS with Applied Biosystems (API2000 LC/MS/MS System, ABI, Foster City, California, USA).

### 2.5. Evaluation of Antioxidant Activity

With 2,2′-azino-bis(3-ethylbenzthiazoline-6-sulfonic acid) (ABTS) assay kit [32, 33], we measured total antioxidant capacity of KA. For preparing the working solution, we mixed two stock solutions of oxidant and ABTS solutions (in equal quantities) and kept the mixed solution at room temperature in the dark for 16 h. Then, we mixed working solution (1 mL) with 80% ethanol (90 mL). Samples (10 *μ*L) were mixed with fresh ABTS working solution (200 *μ*L) at room temperature. After 6 min, the absorbance (at 734 nm) from the mixtures was recorded. We used Trolox as a reference compound. On the other hand, the total antioxidant capacity of KA was evaluated using the assay kit for ferric reducing ability of plasma (FRAP) [34, 35]. We prepared the working solution by mixing detective buffer, TPTZ (2,4,6-tripyridyl-s-triazine), and TPTZ dilution solutions (the stock solutions in a ratio of 1 : 1 : 10). At 37°C, samples (5 *μ*L) were mixed with working solution of FRAP (180 *μ*L). After 5 min, the absorbance (at 593 nm) of the mixtures was recorded. We prepared the standard curve with FeSO_4_. Finally, we showed the values expressed as the mean of 3 measurements.

### 2.6. Animals

Kunming (KM) mice weighing 18–20 g (4 weeks, SPF) were obtained from Beijing HFK Bioscience Co. Inc (Beijing, China). They were placed under standard laboratory conditions (temperature of 24 ± 2°C, relative air humidity of 55 ± 5%, and light/dark cycle of 12/12 h). This study was approved through the Institutional Animal Care and Use Committee from Animal Laboratory Center of Jimei University (SCXK 2016-0006). All experiments met the requirements of the National Laboratory Animal Act (China). According to the principles of the 3Rs (Replacement, Reduction, and Refinement), for decreasing the number of mice used and their suffering, every possible effort into experimental animal design from this study has been adopted. After random grouping, the first group of mice (NC) received the normal chow diet with calorie of 3.42 kcal/g, and the other four groups received HFD (DIO series diets, 60% H10060; calorie, 5.24 kcal/g) [36]. The first group from HFD mice received intragastric gavage (i.g.) of 1.25 mg/kg KA (KAL) (suspended in 0.5% sodium carboxymethyl cellulose (CMC) in phosphate-buffered saline (PBS)). The second group of HFD mice was treated by i.g. of 5 mg/kg KA (KAH) (suspended in 0.5% CMC in PBS). The third group of HFD mice was treated by i.g. of 5 mg/kg SV as positive control group. The last group (HFD mice) and NC group received the same volume of 0.5% CMC in PBS. For 8 weeks, all groups were i.g. administrated by once a day. Kynurenic acid (KA) (catalog no. K3375) was obtained from Sigma-Aldrich (Shanghai) Trading Co., Ltd. (China). We recorded body weight and food intake. All mice were sacrificed with minimize suffering at the end of experiment. After 12 h fasting, serum and fecal samples of mice were collected and then stored at −80°C.

### 2.7. Analysis of Biochemical Parameters

The serum could be obtained by centrifugation from blood for 20 min at 10000 g and at 4°C. TC, TG, LDL-C, and HDL-C levels from serum were determinated with commercially available kits (Mindray, Shenzhen, Guangdong, China) and analyzed by fully automatic bioanalysis machine (BS-240 VET, Mindray Medical International Co., Ltd.) [37]. Coronary artery risk index (CRI) and atherosclerosis index (AI) were counted using following formula [38], respectively:

(1) $$\begin{array}{c} \text{CRI}=\frac{\text{TC}}{\text{HDL}‐\text{C}}, \end{array}$$

(2) $$\begin{array}{c} \text{AI}=\frac{\text{LDL}‐\text{C}}{\text{HDL}‐\text{C}}. \end{array}$$

### 2.8. DNA Extraction and Illumina Sequencing

With a modified method from the previous study [39], we extracted total bacteria DNA of fecal samples by TIANamp Bacteria DNA kit with the manufacturer's instruction (Tiangen Biotech Co., Ltd., Beijing, China). We amplified the V3 and V4 regions from bacterial 16S rRNA with the following primers: 341F (5′-CCTAYGGGRBGCASCAG-3′) and 806R (5′-GGACTACNNGGGTATCTAAT-3′). Following a PE-250 bp sequencing protocol (CapitalBio Technology Co., Ltd., Beijing, China), we sequenced the purified amplicons by a paired-end method using the Illumina Hiseq 2500 platform. After using FLASH (version 1.2.11) and Trimmomatic (version 0.33) for merging and filtering the raw tags from original fragments, the clean tags were obtained. With the UCHIME (version 8.1) software, we removed the chimera sequences for acquiring the effective tags. Using the QIIME (version 1.8.0) software, we clustered tags at 97% similarity level for the operational taxonomic units (OTU). The OUT were classified according to Silva (bacteria) and UNITE (fungi) taxonomic databases. The Mothur (version v.1.30) software was used for analyzing the alpha diversity, including the four indexes of ACE, Chao1, Shannon, and Simpson. Beta diversity was analyzed with the QIIME software, and the principal coordinate analysis (PCoA) chart was drawn basing on R language platform.

### 2.9. Data Analysis

Data in this study were represented as mean ± standard deviation (SD) and further analyzed with Graphpad Prism 6 (GraphPad Software, San Diego, CA, USA). The significance was determined by ANOVA followed by Duncan's multiple-comparison tests. *P* < 0.05 showed a statistically significant difference. *P* < 0.01 and *P* < 0.001 further showed highly significant difference.

## 3. Results

### 3.1. Structure Identification of the Purified Compound KA

**Figure 1:**
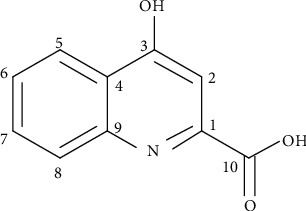
Chemical structure of KA (4-hydroxyquinoline-2-carboxylic acid).

After our calculation, the content of compound KA of the crude extract was about 0.12%. The purity of compound KA was 95%. Compound KA was a white flocculent precipitate, which was soluble in methanol and chloroform and slightly soluble in acetone. The ESI-MS showed signals for quasi-molecular ions with m/z = 187 [M − 2H]^2+^. Based on the 10 carbon signals from the ^13^C-NMR (100 MHz, CDCl_3_) spectrum (Table 1), we concluded a molecular formula of C_10_H_7_NO_3_. *δ*180.5 could be presumed to be the carbon signal on -COOH. *δ*132.3, *δ*139.4, *δ*144.1, and *δ*124.5 could be presumed to be benzene ring the carbon skeleton signal on carbon. *δ*165.4, *δ*108.1, and *δ*118.6 could be presumed to contain -C=C-. The proton ^1^H-NMR spectrum (400 MHz, CDCl_3_) showed 5 proton signals at *δ*7.41 (1H, m), *δ*7.72 (1H, d), and *δ*7.83 (1H, d), which were conjugated to each other. Proton *δ* 8.24 was the -OH proton signal. The ESI-MS and NMR data from KA (Figure 1) were consistent with those reported values from KA in the literature [40].

### 3.2. Antioxidant Activity of KA

**Figure 2:**
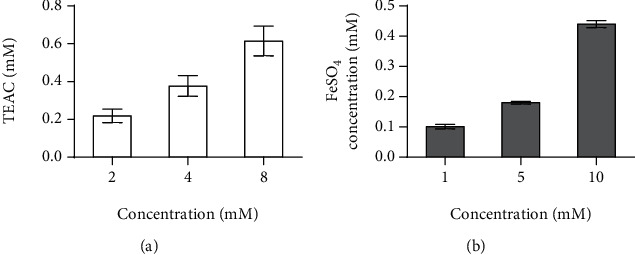
Antioxidant capacity of KA: (a) ABTS method; (b) FRAP method. The antioxidant ability of KA was positively correlated with concentration. All values were represented as mean ± SD (*n* = 3) at 95% confidence intervals.

The ABTS and FRAP assays were applied to evaluate the antioxidant capacities of compound KA (Figure 2). The ABTS and FRAP radical scavenging activities of the KA from horseshoe crab tail were expressed as Trolox equivalents (TE) and FeSO_4_. The higher the concentration of KA, the stronger their ABTS radical cation scavenging capacities (Figure 2(a)). The ABTS values of KA were reached 0.70 mM at concentration of 8 mM. In the FRAP assay (Figure 2(b)), KA exhibited noteworthy ferric reducing antioxidant powers under 10 mM. Antioxidant activities of KA showed dose-dependent effects with the concentration of KA. The FRAP values of KA were reached 0.44 mM at concentration of 10 mM.

### 3.3. Mice Weight and Food Intake

**Figure 3:**
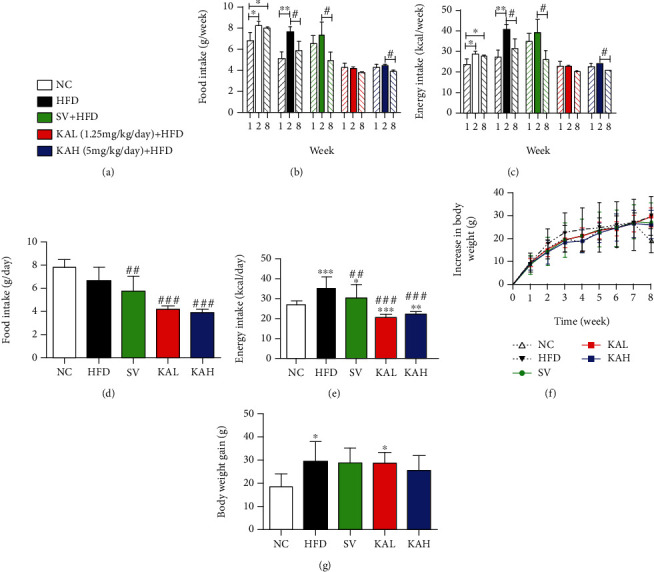
The effects of KA supplementation on food intake, energy intake, and body weight in HFD mice. (a) Classification information for each group. (b) Food intake of per mouse for 1, 2, and 8 weeks (*n* = 3 for NC, HFD, and SV groups; *n* = 6 for KAL and KAH groups). (c) Energy intake of per mouse for 1, 2, and 8 weeks, which was calculated from (b). (d) Food intake of per mouse for each day (*n* = 3 for NC, HFD, and SV groups; *n* = 6 for KAL and KAH groups). (e) Energy intake of per mouse for each day, which was calculated from (d). (f) Increase in body weight (*n* = 5 for NC, HFD, and SV groups; *n* = 6 for KAL and KAH groups). (g) Body weight gain for 8 weeks, which was extracted from (f). KA supplementation reduced food intake, energy intake, and increase in body weight for HFD mice. Data were represented as mean ± SD. ^∗^*P* < 0.05, ^∗∗^*P* < 0.01, and ^∗∗∗^*P* < 0.001 vs. NC mice. #*P* < 0.05, ##*P* < 0.01, and ###*P* < 0.001 vs. HFD mice.

After 8 weeks of feeding experiments, we analyzed the changes in food intake, energy intake, and body weight in mice.  Figure 3(a) is the classification information of each group. Comparing with the NC group in the first week, the weekly food intake (Figure 3(b)) and weekly energy intake (Figure 3(c)) in the NC group increased with the time. In the HFD group, the weekly food intake and weekly energy intake in the second week were significantly higher than those in the first week. The weekly food intake and weekly energy intake in KAL and KAH groups in the second week were still similar with those in the first week, respectively. There was no statistical difference between weekly energy intake of KAL and KAH groups in the first week and that of the NC group in the first week, respectively (Figure 3(c)). We then analyzed the changes in average daily food intake (Figure 3(d)) and average daily energy intake (Figure 3(e)) in mice. Compared with the HFD group, the average daily food intake of SV, KAL, and KAH groups was reduced significantly. Compared with the NC group, the average daily energy intake of HFD and SV groups showed increased significantly and that of KAL and KAH groups showed reduced significantly. Compared with the HFD group, the average daily energy intake of SV, KAL, and KAH groups showed reduced significantly. As the time increased, the body weight in the all groups had an increasing tendency (Figure 3(f)). Compared to the NC group, the body weight gain for 8 weeks of HFD and KAL groups increased significantly (Figure 3(g)). But in the body weight gain for 8 weeks, there was no significant difference between NC and SV or KAH groups, respectively.

### 3.4. Hypolipidemic Effect of KA on HFD Mice

**Figure 4:**
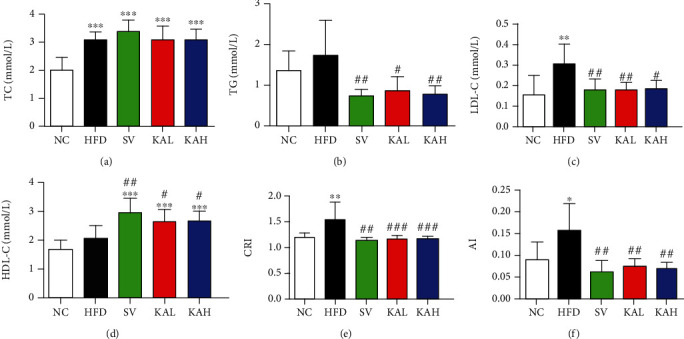
Effects of KA on HFD-caused the changes in blood lipid levels in mice: (a) total cholesterol (TC) in serum; (b) triglyceride (TG) in serum; (c) low-density lipoprotein cholesterol (LDL-C) in serum; (d) high-density lipoprotein cholesterol (HDL-C) in serum; (e) coronary artery risk index (CRI); (f) atherosclerosis index (AI). In HFD mice, KAL and KAH significantly reduced serum TG level and significantly increased serum HDL-C, respectively. KAL and KAH significantly inhibited HFD-induced the increases of serum LDL-C, CRI, and AI levels, respectively. For HFD mice, these effects of KAL and KAH were similar to the effects of SV, respectively. Data were represented as mean ± S.D. (*n* = 5 for each group). ^∗^*P* < 0.05, ^∗∗^*P* < 0.01, and ^∗∗∗^*P* < 0.001 vs. NC mice. #*P* < 0.05, ##*P* < 0.01, and ###*P* < 0.001 vs. HFD mice.

Compared with the NC group, serum TC level in HFD, SV, KAL, and KAH groups was significantly increased (Figure 4(a)). But in TC level, there was no significant difference between HFD group and SV, KAL, or KAH groups, respectively. Compared with the NC group, serum TG level in the HFD group had an upward trend (Figure 4(b)). Compared with the HFD group, serum TG level in SV, KAL, and KAH groups was significantly reduced. Compared with the NC group, serum LDL-C level in HFD group showed increased significantly (Figure 4(c)). Compared to the HFD group, SV, KAL, and KAH significantly reduced HFD-caused the increase in LDL-C level, respectively. But no significant difference for LDL-C level existed between NC group and SV, KAL, or KAH groups, respectively. Compared with the NC group, serum HDL-C from HFD group had an upward trend but that in SV, KAL, and KAH groups were significantly increased, respectively (Figure 4(d)). Compared to the HFD group, HDL-C in SV, KAL, and KAH groups also showed increased significantly, respectively. Further analysis was performed by calculating CRI and AI. Compared with the NC group, CRI level in HFD group increased significantly (Figure 4(e)). Compared to the HFD group, SV, KAL, and KAH significantly inhibited HFD-induced the increase of CRI level, respectively. But no significant difference in CRI level existed between NC group and SV, KAL, or KAH groups, respectively. Compared with the NC group, AI level in HFD group increased significantly (Figure 4(f)). Compared with the HFD group, SV, KAL, and KAH significantly inhibited HFD-induced the increase of AI level, respectively. But no significant difference in AI level existed between NC group and SV, KAL, or KAH groups, respectively.

### 3.5. KA Modulated the Composition in Gut Microbiota of HFD Mice

**Figure 5:**
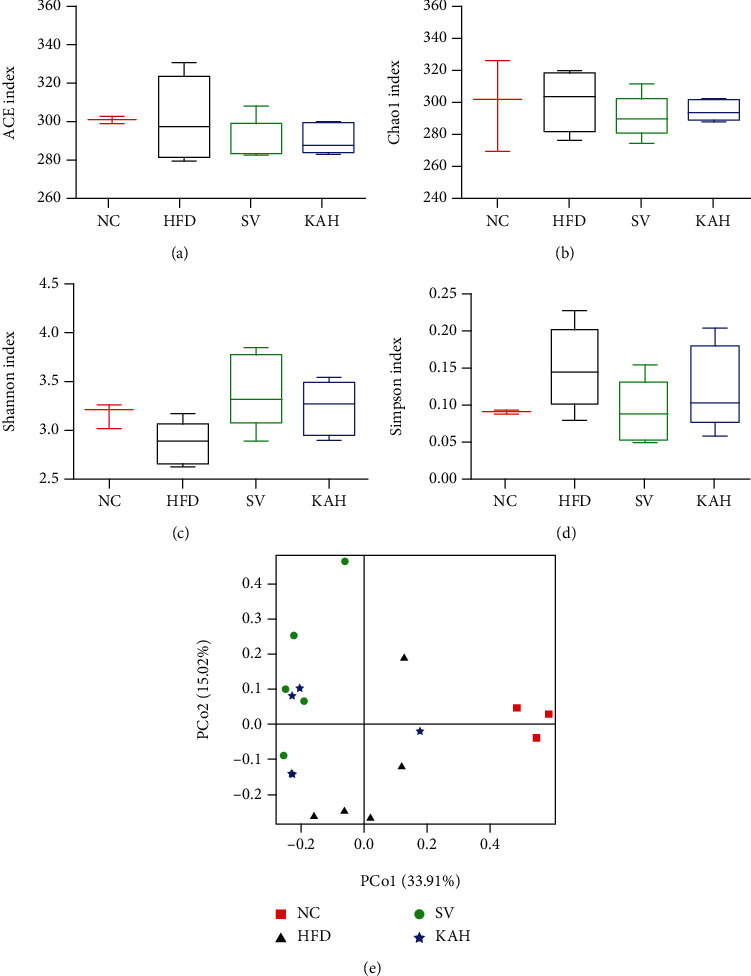
Alpha diversity analysis and beta diversity analysis for gut microbiota: (a) ACE index; (b) Chao1 index; (c) Shannon index; (d) Simpson index; (e) the principal coordinates analysis (PCoA) chart. In alpha diversity analysis, no significant differences at *P* > 0.05 (a–d) were observed between NC group and HFD, SV, or KAH groups, respectively. In beta diversity analysis, neither SV nor KAH obviously reversed the changes induced by HFD (e). Data were represented as mean ± SD for (a–d) (*n* = 3 for NC group, *n* = 5 for other groups).

**Figure 6:**
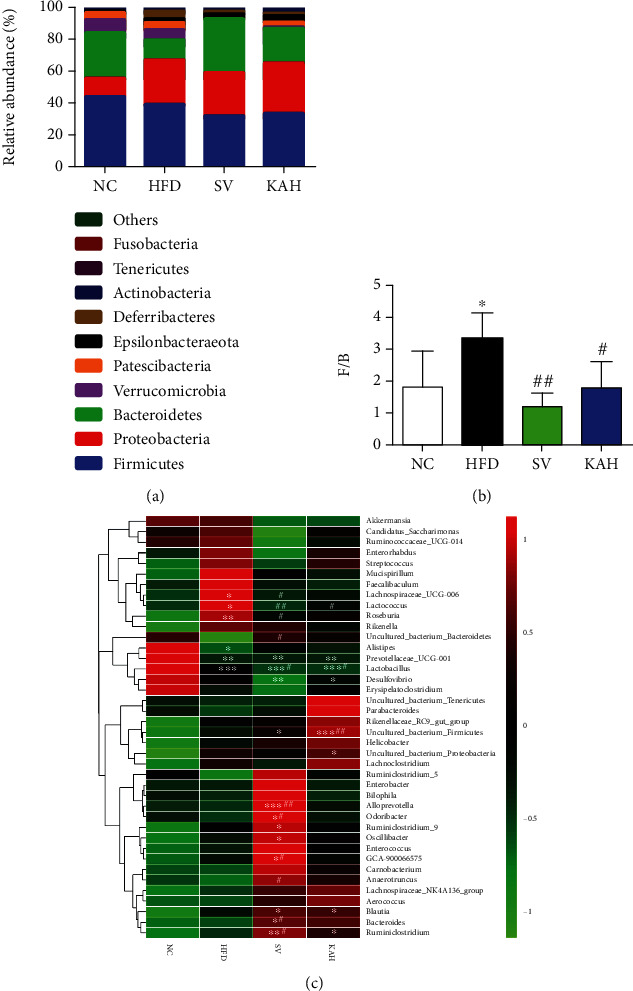
Supplementation of KA modulated the composition in gut microbiota: (a) percent of community abundance at phylum level among the NC, HFD, SV, and KAH groups; (b) the ratio of Firmicutes to Bacteroidetes (F/B ratio); (c) heatmap analysis with the genus level for NC, HFD, SV, and KAH groups. There were marked differences among the groups in the level of the two microbial phyla, Firmicutes and Bacteroidetes, which showed dominant for the gut microbial composition. KAH significantly inhibited HFD-induced the increase of F/B ratio. For HFD mice, the effect of KAH on F/B ratio was similar to that of SV. Compared to NC group, KA reversed HFD-caused the changes in some microbial genera. Data were represented as mean ± SD (*n* = 3 for NC group, *n* = 5 for other groups). ^∗^*P* < 0.05, ^∗∗^*P* < 0.01, and ^∗∗∗^*P* < 0.001 vs. NC mice. #*P* < 0.05 and ##*P* < 0.01 vs. HFD mice.

We collected 1,440,504 high-quality sequences in the 16S rRNA (the V3-V4 region) from 18 fecal samples after 8 weeks for understanding the effect of the KA on the composition in gut microbiota of HFD mice. We showed effective tags from sequencing data in Supplementary Table S1 and rarefaction curves in Supplementary Figure S1. We found that the average sequence number was 72,196 (the minimum was 70,541, and the maximum was 74,037), and the average length was 420 bp. By deleting the reads with low quality, we reserved 1,174,687 operational taxonomic units (OTUs) with the similarity level of 97%. No significant differences at *P* > 0.05 in alpha diversity analysis (Figures 5(a)–5(d)) were observed between NC group and HFD, SV, or KAH groups, respectively. Neither SV nor KAH obviously reversed the changes in beta diversity analysis caused by HFD (Figure 5(e)). Taxon-based analysis showed that KA supplementation caused obvious changes in the gut microbial composition. We observed marked differences among the groups in the level of two microbial phyla, Firmicutes and Bacteroidetes, which showed dominant for the gut microbial composition (Figure 6(a)). Compared to the NC group, the ratio of Firmicutes to Bacteroidetes (F/B ratio) from the HFD group increased significantly (Figure 6(b)). Compared to the HFD group, SV and KAH significantly inhibited HFD-caused the increase in F/B ratio, respectively. But no significant difference in F/B ratio existed between NC group and SV or KAH groups, respectively. Additionally, our further analysis with lower taxonomic level (genus) showed that the effect of KAH on relative abundance in 40 key genera of HFD mice (Figure 6(c)). Compared with the NC group, HFD significantly increased the abundance of *Lachnospiraceae_UCG-006*, *Lactococcus*, and *Roseburia*; HFD significantly reduced the abundance of *Alistipes*, *Prevotellaceae_UCG-001*, and *Lactobacillus*. Compared to the HFD group, SV and KAH modulated some microbial genera. SV and KAH significantly inhibited HFD-induced the increase of the abundance of *Lachnospiraceae_UCG-006*, *Lactococcus*, and *Roseburia*. SV and KAH significantly attenuated HFD-induced the decrease of the abundance of *Alistipes*. But SV and KAH did not affect HFD-caused the decrease of the abundance of *Prevotellaceae_UCG-001* and significantly enhanced HFD-induced the decrease of the abundance of *Lactobacillus*. Compared to the NC group, SV and KAH significantly reduced the abundance of *Desulfovibrio* and significantly increased the abundance of *uncultured_bacterium_Firmicutes*, *Blautia*, and *Ruminiclostridium*. In addition to the same effects of SV and KAH mentioned above, there were also the following different effects of SV and KAH. Compared to the HFD group, SV significantly increased the abundance of *uncultured_bacterium_Bacteroidetes*. Compared to the NC group, SV significantly increased the abundance of *Alloprevotella*, *Odoribacter*, *Ruminiclostridium_9*, *Oscillibacter*, *GCA-900066575*, *Anaerotruncus*, and *Bacteroidies*. Compared with the NC group, KAH significantly increased the abundance of *uncultured_bacterium_Proteobacteria*.

## 4. Discussion

### 4.1. The New Findings of This Study

In this study, we report six new findings, as compared with a previous study about KA in 2018 [20]. First, NMR and ESI-MS were used to identify the compound isolated from the horseshoe crab (*Tachypleus tridentatus*) tail, and it was determined to be KA (Figure 1). Second, i.g. of 5 mg/kg/day KA (KAH) significantly inhibited HFD-induced body weight gain in mice (Figure 3(g)). This previous study has reported that the i.p. administration of KA (5 mg/kg/day) prevents body weight gain induced by HFD in mice, and duration of administration of KA in this experiment is 4 weeks [20]. Additionally, we observed that i.g. of 5 mg/kg/day KA (KAH) for 8 weeks did not produce any obvious side effects in general appearance of HFD mice. Third, the inhibitory effect of KAH on HFD-induced body weight gain was similar to that of SV, a positive control. And, we selected two dosages of KA, 1.25 mg/kg/day KA (KAL) and 5 mg/kg/day KA (KAH), for HFD mice in this study. Although this previous study selected only one dosage of KA, 5 mg/kg/day, for HFD mice [20]. Fourth, KAL and KAH significantly reduced average daily food intake in HFD mice (Figure 3(d)) and significantly inhibited HFD-induced the increase of average daily energy intake (Figure 3(e)), respectively. Although this previous study has reported that i.p. administration of KA (5 mg/kg/day) for 3 weeks did not cause the change in food intake of the normal mice [20]. Fifth, in HFD mice, KAL and KAH significantly reduced serum TG level (Figure 4(b)) and significantly increased serum HDL-C (Figure 4(d)), respectively. KAL and KAH significantly inhibited HFD-induced the increases of serum LDL-C (Figure 4(c)), CRI (Figure 4(e)), and AI (Figure 4(f)), respectively. For HFD mice, these effects of KAL and KAH were similar to the effects of SV, respectively. Sixth, KAH significantly inhibited HFD-induced the increase of F/B ratio, the ratio of two dominant gut microbial phyla (Figures 5(a) and 5(b)). For HFD mice, the effect of KAH on F/B ratio was similar to that of SV. Compared with the NC group, KAH partially reversed HFD-induced the alterations in the composition of gut microbial genera (Figure 6(c)).

### 4.2. The Effects of KA on HFD-Induced Obesity and Hyperlipidemia

In 2020, Li et al. indicated that KM mouse accounted for more than 70% of experimental mice in China for both biology and medical studies, and KM mouse was one of widely used mouse strains for obese model induced by HFD [41]. We found that HFD indeed caused the significant obesity characteristics (Figure 3) and hyperlipidemia (Figures 4) in KM mice compared with the NC group. Hyperlipidemia has been identified as a pivotal hazard element for the formation and progress of coronary heart disease and atherosclerosis. Continuous excessive ingestion of lipids is one of important reasons for leading to the harmful cycle for lipid metabolism [42]. The decrease levels of TC or TG could be capable to prevent or treat hyperlipidemia. LDL-C has been indicated to be a crucial transporter for TC [43]. The easy cumulation of redundant LDL-C of blood vessels could be oxidized, which enhance the generation of both foam cells and plaques [44]. KAL and KAH significantly inhibited HFD-induced the increases of serum LDL-C, respectively (Figure 4(c)). Although in HFD-induced the increase of serum TC level, we found that no significant difference between HFD group and SV, KAL, or KAH groups, respectively (Figure 4(a)). On the other hand, with the “reverse cholesterol transport” pathway, the cholesterol of tissues or blood vessels could be transported into the liver via HDL-C, and it could be cleared through bile acids as well as excretion [45, 46]. KAL and KAH significantly increased serum HDL-C (Figure 4(d)) in HFD mice, respectively. Moreover, KAL and KAH significantly inhibited HFD-induced the increases of serum CRI (Figure 4(e)) and AI (Figure 4(f)), respectively. Based on the present results, we suggest that KA has the hypolipidemic effect and cardiovascular protective function.

### 4.3. The Possible Mechanisms of i.g. of KA on HFD Mice

**Figure 7:**
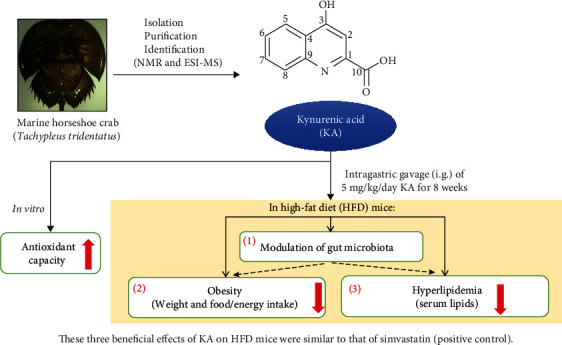
The beneficial effects of oral supplementation of KA from marine horseshoe crab on HFD mice. This study isolated KA from horseshoe crab in for the first time in the world. We found that KA (5 mg/kg/day) inhibited both the body weight gain and the increase of average daily energy intake in HFD mice. We suspect that the improvement effects of KA on HFD-induced obesity and hyperlipidemia may be through the modulation of gut microbiota. Our findings demonstrate the potential therapeutic effects of KA for HFD mice, which were similar to that of simvastatin (positive control). The *in vitro* results suggest that the improvement effects of KA on HFD mice may be partially associated with antioxidant activity of KA.

KA does not cross the blood-brain barrier easily under normal conditions and does not have a significant effect on the central nervous system [47]. Therefore, the exogenous oral supplementation of KA could be considered to have the major effects on the peripheral system [48]. A previous study demonstrated that KA enhances energy utilization through activation of Gpr35, which regulates adipose tissue, including stimulating expressions of lipid metabolism, thermogenesis, and anti-inflammation-related genes [20]. However, based on our experimental results, we speculate that the beneficial effects of i.g. of KA on HFD mice should also include other mechanisms (Figure 7). According to a previous study in mice, HFD may cause massive free radicals and also inhibit the antioxidant protection system [49]. The reactive oxygen species (ROS) were reported to oxidize LDL-C for forming ox-LDL-C that could be highly related with the development of hyperlipidemia [50]. In the ABTS test, KA could scavenge the free radicals, and the effect of scavenging the free radicals is increased by increasing the concentration (Figure 2(a)). In the reducing power assay, the reducing power of KA increases by increasing concentration (Figure 2(b)). The ABTS free radical scavenging ability and reducing capacity of KA showed its potential antioxidant activity, which is consistent with a previous study [13]. KA (2.5 mg/L in drinking water) significantly decreased the oxidative burst intensity from phagocytes in mice, which could exert protection from oxidative stress [51]. On the other hand, Firmicutes and Bacteroidetes among the groups showed dominant within the composition of gut microbiota (Figure 6(a)). This previous study showed the phenomenon of increase of the intestinal F/B ratio from obesity [52], which is supported by our data. F/B ratio in the HFD group increased significantly compared with the NC group, and KAH significantly inhibited HFD-induced the increase of F/B ratio (Figure 6(b)). Additionally, our further analysis with the lower taxonomic level (genus; Figure 6(c)) revealed that some microbial genera may have the roles in the beneficial effects of KA for HFD mice. The previous study reported that the family *Lachnospiraceae* might be linked to development of obesity and metabolic disorders [53]. Compared with the NC group, HFD increased the abundance of *Lachnospiraceae_UCG-006* (Figure 6(c)), which is consistent with a previous study [54]. A previous study in mice also demonstrated that HFD caused the elevated abundance of *Lachnospiraceae_UCG-006* and further showed that the deteriorated lipid profiles were positively correlated with the abundance of *Lachnospiraceae_UCG-006* [55]. In mice, HFD was found to increase the abundance of *Lactococcus*, and *Lactococcus* was reported to be positively correlated with serum dyslipidemia [56]. Both SV and KAH significantly inhibited HFD-induced hyperlipidemia (Figure 4) and the increase of the abundance of *Lachnospiraceae_UCG-006* and *Lactococcus* (Figure 6(c)). Based on the previous studies and our present results, we suggest that the beneficial effects of oral supplementation of KA on HFD mice may be partially associated with its inhibitory effects on the increase of the abundance of *Lachnospiraceae_UCG-006* and *Lactococcus*. But the possibility that other microbial genera could also contribute to the beneficial effects of oral KA on HFD model cannot be excluded. KA could also had tendency to increase the abundance of some gut microbiota genera, which were negatively associated with obesity (Figure 6(c)), e.g., *Bacteroides* [57]. Therefore, this study suspects that the beneficial effects from KA on HFD-induced obesity and hyperlipidemia may also be mainly associated with its antioxidant activity and ability for modulation of gut microbiota.

### 4.4. The Characteristics of Oral Supplementation of KA for the HFD Model

Compared with the NC group, we found that HFD caused the significant obesity (Figure 3(g), *P* < 0.05), hyperlipidemia (Figures 4(a) (*P* < 0.001), 4(c) (*P* < 0.01), 4(e) (*P* < 0.01), and 4(f) (*P* < 0.05)), and changes of gut microbiota (Figures 6(b) (*P* < 0.05) and 6(c) (from *P* < 0.05 to *P* < 0.001 for 6 microbial genera, respectively)) in mice. In this study, there were statistically significant differences for the above three phenomena between KA and HFD groups, which supported the finding that the beneficial effects of oral supplementation of KA on HFD mice. But certainly, the detailed cellular mechanisms of oral KA on HFD model still need to be further explored in future study. The i.g. of 5 mg/kg/day KA (KAH) for 8 weeks did not cause any obvious side effects in general appearance of HFD mice, which is consistent with two previous reports [58, 59]. Dietary supplementation of KA (250 mg/L in drinking water) did not induce toxic effects in normal adult rats (continuous supplementation for 21 days) or normal adult mice (continuous supplementation for 14 days) [59]. Chronic dietary supplementation with KA (25 mg/L or 250 mg/L in drinking water) for 40 days decreased body weight but without negative influence on the development of the skeleton in young normal rats [58]. Importantly, several products for the daily human diet were also found to contain KA, including broccoli, potato, honeybee products, tea, and coffee [48, 60]. These previous studies and the results of this study provide the safety basis for KA to be used as food ingredient. Furthermore, KA has been reported to be absorbed by the intestine and achieve high concentrations in the liver and kidneys [48]. Another previous study also demonstrated that intragastrical administration of KA could be absorbed by the digestive system and further excreted through urine [60]. These previous studies provide the scientific basis for the use of KA via the oral route. This study also demonstrates three characteristics of KA for the HFD model. First, the inhibitory effect of KA on HFD-induced body weight gain was similar to that of SV, a positive control (Figure 3(g)). Second, the effects of 1.25 mg/kg/day KA on HFD-caused hyperlipidemia were similar to the effects of 5 mg/kg/day SV (Figure 4). Third, the improvement effects of KA on HFD-induced the changes in gut microbiota were similar to that of SV (Figure 6), but the pattern of relative abundance in 40 key genera from the KA group was closer to that from the NC group than that from the SV group (Figure 6(c)).

## 5. Conclusions

Our findings demonstrate KA has a potential functional food ingredient role for treating obesity and hyperlipidemia as well as the modulation of gut microbiota. This study, involving identification of KA as a bioactive component from the horseshoe crab, also helps to find substitutes for these creatures, which will promote the conservation of these creatures.

## Figures and Tables

**Table 1:** The NMR data and C and H assignment of compound KA.

No.	^13^C	^1^H	^1^H-^1^H COSY	HMBC
1	165.4	—	—	—
2	108.1	6.97 (1H, s)	—	C-1, C-5, C-7
3	118.6	—	—	—
4	139.4	—	—	—
5	132.3	7.41 (1H, m)	H-6, H-8	C-3, C-5, C-8
6	139.4	7.72 (1H, d)	H-5, H-7	C-8, C-9
7	146.1	7.83 (1H, d)	H-6, H-8	C-8
8	124.5	7.41 (1H, d)	H-5, H-6	C-5, C-9, C-10
9	146.1	—	—	—
10	180.5	—	—	—

## Data Availability

Data available on request. We make data available on request through the corresponding authors (Shi-Ying Huang, E-mail: johnhuang@jmu.edu.cn; Hui-Min David Wang, E-mail: davidw@dragon.nchu.edu.tw).

## Supplementary Materials

Supplementary materials Supplementary Table S1: sequencing data for the 16S rRNA from fecal samples of mice. Supplementary Figure S1: rarefaction curves from sequencing data for the 16S rRNA from fecal samples of mice.
