# Supplementary material for: The Beneficial Effects of Edible Kynurenic Acid from Marine Horseshoe Crab (Tachypleus tridentatus) on Obesity, Hyperlipidemia, and Gut Microbiota in High-Fat Diet-Fed Mice
Source: Oxid Med Cell Longev. 2021 May 3;2021:8874503. doi: 10.1155/2021/8874503 (PMC8112934; doi:10.1155/2021/8874503)
Supplement: Supplementary materials — Supplementary Table S1: sequencing data for the 16S rRNA from fecal samples of mice. Supplementary Figure S1: rarefaction curves from sequencing data for the 16S rRNA from fecal samples of mice. [file 8874503.f1.docx]

**Supplementary Materials**

**Supplementary Table S1.** Sequencing data for the 16S rRNA from fecal samples of mice.

| Sample ID | PE Reads | Clean Tags | Effective Tags | Effective (%) |
| --- | --- | --- | --- | --- |
| NC1 | 79939 | 63996 | 57643 | 72.11 |
| NC2 | 79914 | 65376 | 60211 | 75.34 |
| NC3 | 79758 | 64233 | 61061 | 76.56 |
| HFD1 | 79911 | 63339 | 62060 | 77.66 |
| HFD2 | 80310 | 65467 | 62492 | 77.81 |
| HFD3 | 80244 | 65306 | 62087 | 77.37 |
| HFD4 | 80376 | 66463 | 63828 | 79.41 |
| HFD5 | 79883 | 67585 | 62764 | 78.57 |
| SV1 | 80005 | 65852 | 63406 | 79.25 |
| SV2 | 80023 | 63844 | 60812 | 75.99 |
| SV3 | 79908 | 64276 | 61219 | 76.61 |
| SV4 | 79923 | 65098 | 63444 | 79.38 |
| SV5 | 80475 | 67743 | 65067 | 80.85 |
| KAH1 | 80119 | 65263 | 61968 | 77.34 |
| KAH2 | 79854 | 64905 | 61031 | 76.43 |
| KAH3 | 80017 | 64874 | 63522 | 79.39 |
| KAH4 | 79909 | 65800 | 63552 | 79.53 |
| KAH5 | 79936 | 65267 | 63268 | 79.15 |
| Sum total | 1440504 | 1174687 | 1119435 |  |

Sample ID: the name of the sample from each mouse. PE Reads: the number of paired-end reads obtained by sequencing. Clean Tags: the number of optimized sequences obtained by filtering the raw sequences. Effective Tags: the number of sequences obtained by filtering the chimera from clean tags. Effective (%): the percentage of effective tags in PE Reads.


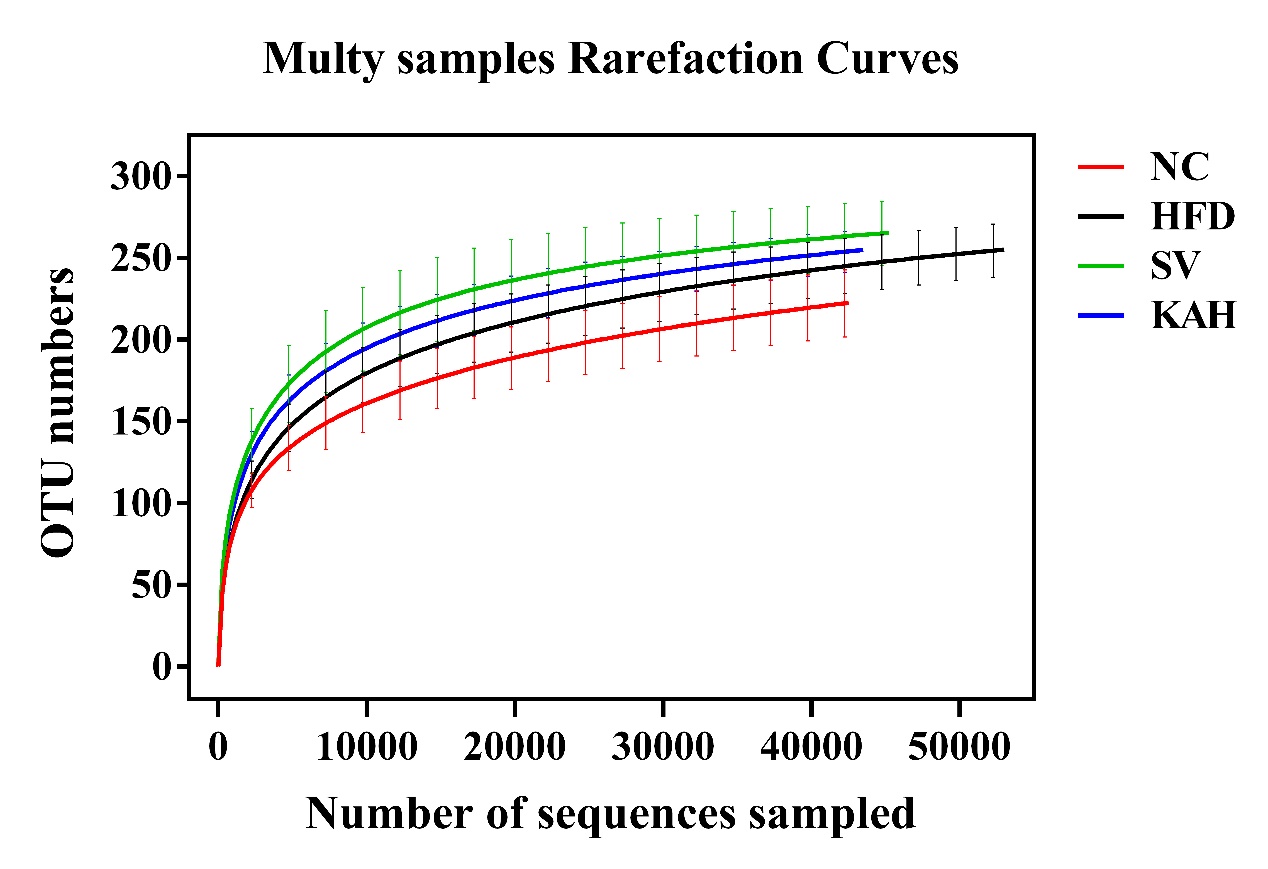


**Supplementary Figure S1. Rarefaction curves from sequencing data for the 16S rRNA from fecal samples of mice.** Data were represented as mean ± SD (*n* = 3 for NC group, *n* = 5 for other groups).
